# The Location of the Deepest Point of the Eyeball Determines the Optic Disc Configuration

**DOI:** 10.1038/s41598-017-06072-8

**Published:** 2017-07-19

**Authors:** Yong Chan Kim, Younhea Jung, Hae-Young Lopilly Park, Chan Kee Park

**Affiliations:** Department of Ophthalmology, Seoul St. Mary’s Hospital, College of Medicine, The Catholic University of Korea, Seoul, Korea

## Abstract

Tilted and rotated appearances are hallmarks of the myopic optic disc. As the eyeball grows axially, the posterior pole elongates not only globally but in a localized manner as well. In this process, the optic disc is pulled towards the deepest point of the elongated eyeball, which might result in a change in optic disc configuration. Thus, we hypothesized that analyzing the variation of posterior pole contour can play a major role in understanding optic disc configuration in myopic subjects. By analyzing consecutive images of swept source OCT coronal sections at the posterior pole, the deepest interface between Bruch’s membrane and the choroid could be identified as the deepest point of the eyeball (DPE). The location and the properties of the DPE differed significantly between the 125 eyes of non-glaucomatous myopic group and the 40 eyes of non-glaucomatous emmetropic group classified based on 24 mm axial length. The results suggested that the larger disc to DPE angle and the larger disc to DPE depth strongly predicts the optic disc torsion degree and the optic disc tilt. Our findings suggest that identifying the posterior pole profile plays a major role in understanding the optic disc alterations found in myopic subjects.

## Introduction

Epidemiological studies conducted in various locations have consistently shown myopia to be a risk factor for glaucoma^1, 2^. Since there has been a notable increase in the prevalence of myopia in recent date, proper assessment of the increasing number of myopic patients has become more important^3, 4^. The higher susceptibility to glaucoma is hypothesized by conformational changes induced by post-natal axial elongation of the eyeball in the posterior direction^5^. A variety of conformational changes have been documented in myopic eyes such as, enlargement and flattening of the optic disc^6^, lamina cribrosa and peripapillary scleral thinning^7^, skewed or unusually large scleral canal^8^, some of which are believed to be partly responsible for increased susceptibility to glaucoma.

Tilted and rotated appearances are hallmarks of the myopic optic disc. It has been hypothesized that a tilted disc originates from oblique insertion of the optic nerve at birth^9^. On the other hand, recent study showed a progressive tilting of the optic disc in children with incipient myopia^10^. The association of the myopic shift along with the development of tilted and rotated disc suggests that disc configuration change is the result of posterior scleral remodeling associated with axial elongation of the eyeball. In histologic findings of axial elongation, density of retinal pigment epithelium cells (RPE) and thickness of retina is distinct by location, showing decreased RPE density and retinal thickness in the retro-equatorial region of the sclera in comparison with the posterior pole^11, 12^. It led to the hypothesis that axial elongation occurs in the retro-equatorial region which would result a tube-like enlargement of the globe rather than spherical enlargement^13^. In this process, there must be an asymmetric growth in the retro-equatorial region of the globe thus resulting in a winding tube-like enlargement. Thus, we hypothesized that the winded eyeball would pull the optic disc toward the deepest point of the eyeball, which might result in an optic disc configuration alteration such as optic disc tilt or torsion.

Previously, our group imaged the sclera at the posterior pole using swept source optical coherence tomography (SSOCT) and measured the subfoveal scleral thickness in glaucoma patients^14^. The subsequent study revealed that the optic disc tilt and torsion was significantly related with the location of the thinned sclera^15^. These findings brought about the necessity for the precise evaluation of the posterior pole profile and further investigate its correlation with the optic disc configuration. En face OCT is one of the OCT visualization approaches that has significantly benefited from technical advancements in OCT technology^16^. While clinicians are used to viewing high-resolution OCT cross-sectional scans, en face OCT takes a different approach. After using a dense raster scan to acquire an image cube of the posterior pole, software is used to reconstruct a coronal view of the eyeball that is sectioned into ultrathin 2.6 μm in the anteroposterior orientation^17^. En face OCT provides numerous advantages, not only the ability to precisely localize lesions within specific subretinal layers, also the ability to register projected OCT images to other fundus imaging modalities because it shares the same plane as the common fundus photograph. In the glaucoma circles, there are various attempts to utilize en face images, such as the measuring local retinal nerve fiber layer (RNFL) thickness^18^. Furthermore, SSOCT uses longer wavelength which enables to clearly visualize the deeper structures of the eyeball such as the choroid and the posterior sclera. We hypothesized that by analyzing the consecutive images of coronal section around the posterior pole, the deepest interface between Bruch’s membrane and choroid could be identified as a decreasing plane which indicates the deepest point of the eyeball (DPE). Once the location of precise DPE in the posterior pole is located, we could apply the location in the common fundus photograph, thus enabling the precise relationships between the DPE and the optic disc tilt and torsion.

In the present study, we analyzed the relationship between the DPE and optic disc tilt and torsion in myopic non-glaucomatous eyes and compared it with the emmetropic non-glaucomatous eyes. Moreover, we subdivided the myopic group by the different location of the DPE, and the comparison between the 4 different groups was evaluated to find out if there are specific properties of each group.

## Methods

This retrospective, observational study was approved by the Institutional Review Board of Seoul St. Mary’s Hospital, which followed the principles of the Declaration of Helsinki.

### Study Participants

We enrolled patients evaluated at the glaucoma clinic of the Seoul St. Mary’s Hospital between September 2016 and January 2017 and informed consent was obtained. Each subject received a comprehensive ophthalmologic assessment, including a review of the medical history, measurement of best-corrected visual acuity (BCVA), refraction, slit-lamp biomicroscopy, gonioscopy, Goldmann applanation tonometry, dilated stereoscopic examination of the optic disc and fundus, color disc photography, red-free retinal nerve fiber layer (RNFL) photography (VX10; Kowa Optimed, Tokyo, Japan), and achromatic automated perimetry using the Swedish Interactive Threshold Algorithm standard 24-2 test (Humphrey Visual Field Analyzer; Carl Zeiss Meditec, Inc., Dublin, CA, USA). The central corneal thickness and axial length of each eye were also measured during the initial presentation via ultrasound pachymetry (Tomey Corporation, Nagoya, Japan) and ocular biometry (IOL Master; Carl Zeiss Meditec, Inc., Dublin, CA, USA). The retinal nerve fiber layer (RNFL) thickness was measured by intrinsic program in the SSOCT (DRIOCT Triton, Topcon corporation, Tokyo, Japan).

To be included in the present study, eyes had to have an open iridocorneal angle on gonioscopic examination. To minimize the effect of media opacity, the corrected visual acuity was better than 20/40. Subjects had to have a baseline intraocular pressure (IOP) of less than 21 mm Hg without anti-glaucoma medication, absence of glaucomatous optic disc changes such as localized or diffuse rim thinning and retinal nerve fiber defects, absence of glaucomatous visual field (VF) defects corresponding to the glaucomatous structural changes. Glaucomatous VF defects were defined by glaucoma hemifield test results outside normal limits or the presence of at least three contiguous non-edge test points within the same hemifield on the pattern deviation plot at <5%, with at least one of these points at <1%, confirmed by two consecutive reliable tests (false negative <15%, false positive <15%, and fixation losses <20%).

The subjects were assigned to myopia group if they had an axial length >24.0 mm and spherical equivalent (SE) <−2D. The subjects were assigned to emmetropic group if they had an axial length <24.0 mm and SE >0.5D; if the eye was pseudophakic or had refractive surgery, pre-operative SE was used to evaluate the eligibility.

The subjects were excluded if they had a history or evidence of glaucoma or other optic neuropathies, congenital anomalies of the optic disc, an history of elevated IOP, signs of pathologic myopia including myopic choroidal neovascularization, lacquer crack, angioid streak. To fulfill our purpose which was to identify the primary association between posterior pole profile and optic disc configuration and compare it in myopic and emmetropic subjects, eyes with an axial length over 30 mm was also excluded. In cases in which both eyes of a subject were eligible for the study, only one eye was randomly chosen for inclusion.

### En Face Analysis of SSOCT Scans

**Figure 1:**
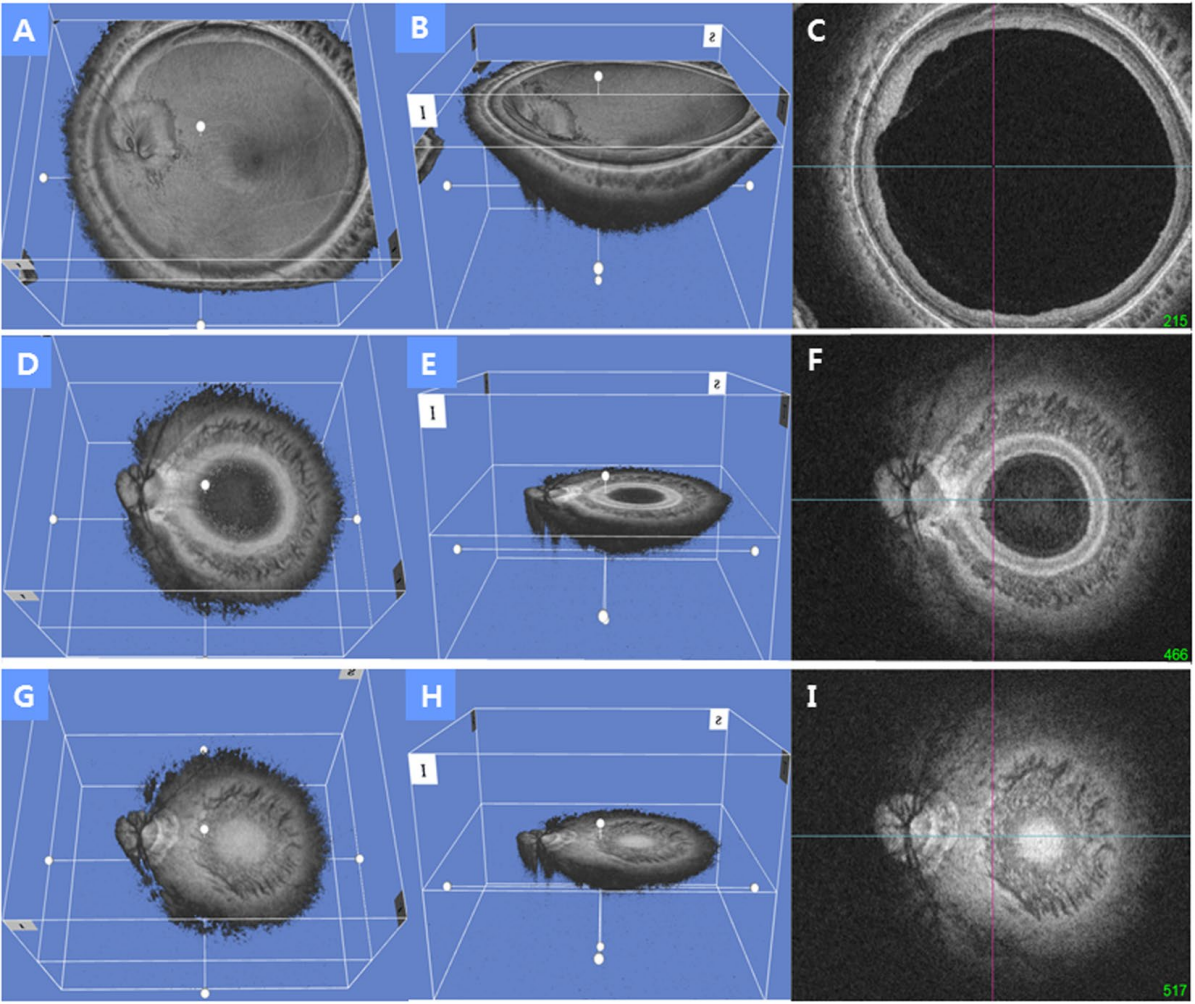
Representative three-dimensional tomogram of the posterior pole by swept-source optical coherence tomography. (**A**) Section of the eyeball on the nasal border of the optic disc. (**B**) Oblique view of the same section as A. (**C**) En face view of the same section as A. (**D**) Section of the eyeball on the temporal border of the optic disc. The displayed section is moved in the posterior direction from A. (**E**) Oblique view of the same section as D. (**F**) En face view of the same section as D. (**G**) Section of the eyeball on the posterior pole. The interface of Bruch’s membrane in the image appears as a hyper-reflective round plane that is surrounded by the choroid, indicating the deepest point of the eyeball (DPE) of the posterior retinal contour. The displayed section is moved in the posterior direction from D. (**H**) Oblique view of the same section in G. (**I**) En face view of the same section as G. This interface was defined as the DPE of the posterior pole contour.

The detailed specification of the ssOCT were described previously^19^. To sum up, ssOCT (DRIOCT Triton, Topcon corporation, Tokyo, Japan) has a scan speed of 100,000 A-scans/sec and center wavelength of the light beam is 1050 nm and the effective axial resolution is 8 μm in tissue. The reference mirror was placed at a deeper position of the retina so that the sensitivity was higher at the choroid and sclera. En face, or C-scan, images were obtained with a prototype software provided by Topcon (Topcon Corporation, Tokyo, Japan) which generates the images after an automatic averaging and flattening at RPE level, using the three dimensional volumetric scan of 12 × 9 mm, yielding a tissue imaging depth of 2.6 mm which comprised of 1,000 coronal sections (Fig. 1)^20^. This segmentation allows a better quality of the images due to the correction of the distortions. En face mode provides a coronal view of the posterior segment at different depth levels. It supplies additional information to conventional cross-sectional imaging, allowing the physicians to access the posterior segment beyond the RPE level, in a non-invasive manner.

### Measurement of the Deepest Point of the Eyeball

**Figure 2:**
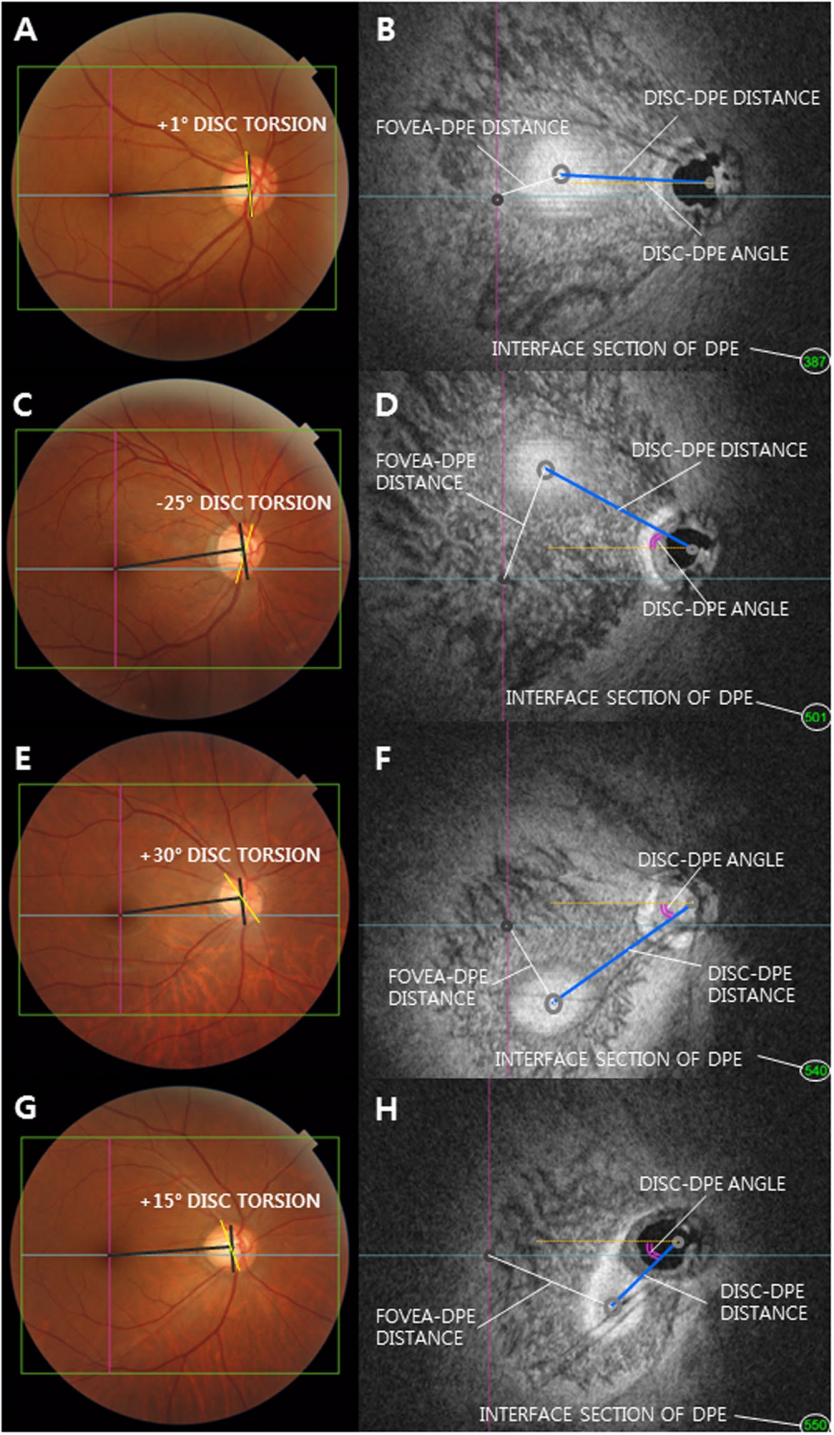
Classification of the deepest point of the eyeball (DPE) by location in the coronal plane. (**A**,**C**,**E**,**G**) Optic disc photography measuring the optic disc torsion degree. Disc torsion was identified and defined as the deviation of the long axis of the optic disc (yellow line) from the vertical meridian (dark gray line). (**B**) En face view of A. Schematic diagram of quantifying the DPE by relative position in the coronal section. Eyes that had the center of the DPE within 3000 μm of the fovea were categorized as the near fovea group. (**D**) En face view of C. Eyes that had the center of the DPE in the superior half of the globe, based on an imaginary line connecting the fovea and the center of the disc and excluding the fovea and disc area, were categorized as the superior hemisphere group. (**F**) En face view of E. Eyes that had the center of the DPE in the inferior half of the globe, based on the imaginary line connecting the fovea and the center of the disc and excluding the fovea and disc area were categorized as the inferior hemisphere group. (**H**) En face view of G. Eyes that had the center of the DPE within 3000 μm of the disc were categorized as the near disc group.

Each en face image is the coronal view of the eyeball that is sectioned into ultrathin 2.6 μm in the anteroposterior orientation. By analyzing the consecutive images of coronal section around the posterior pole, the interface between the different tissues can be identified by the difference of the reflectivity between the two separate tissues. The interface of the Bruch’s membrane in the en face images appears as the hyper-reflective round plane that is surrounded by the choroid, indicating the deepest point of the eyeball (DPE) (Figs 1 and 2). The examiner moved the coronal sections back and forth to spot the exact interface between the Bruch’s membrane and the choroid tissue. This interface was defined as the DPE of the posterior pole contour. Although the outer integument of the eyeball is the sclera, the retinal conformation is in line with the Bruch’s membrane than the choroid or the sclera. So the measurements of the DPE relative position were performed in the coronal plane of the Bruch’s membrane/Choroid interface.

We quantified the DPE location in the posterior pole by using the caliper function of the built-in software of the OCT by 2 authors in a blinded fashion (YCK and HYP). The SSOCT software provides center of the disc as a green cross based on the margin of Bruch’s membrane as a default. The Disc-DPE distance was defined as the straight line distance between the center of the optic disc to the center of the DPE measured at the same coronal plane of the DPE. Likewise, the Fovea-DPE distance was defined as the straight line distance between the fovea to the center of the DPE measured at the same coronal plane of the DPE. To quantify the angular position of the DPE relative to the optic disc, the horizontal meridian crossing the OCT-defined center of the optic disc was determined as the reference line. The Disc-DPE angle was defined as the angle between the horizontal meridian crossing the OCT-defined center of the optic disc and the straight line from the OCT-defined center of the optic disc to the DPE measured by the intrinsic caliper of the built-in software. Likewise, the Disc-Fovea angle was defined as the angle between the horizontal meridian crossing the OCT-defined center of the disc and the straight line from the OCT-defined center of the optic disc the fovea measured by the intrinsic caliper of the built-in software^21, 22^. To compensate for the potential errors induced by head tilt or ocular rotation, the subjects were seated at the fundus camera with their chin in the chin rest and forehead against the forehead rest. The subjects’ eyes were aligned with the eye level mark on the forehead rest support by raising or lowering the chin rest. They were instructed to hold their heads in a vertical position throughout the photographic session. Using the eye to be photographed, each patient was instructed to look directly at the internal fixation target in the OCT camera, which was used as a marker for the foveal center. The depth between the different posterior pole contour structures was calculated by the number of coronal sections between the interfaces of the different structures. After counting the depth by the separate images of the coronal section, the depth was switched into micrometers by counting each coronal section as 2.6 μm in depth. The Disc-DPE depth was the depth between the interface of the DPE and the interface of temporal border of the optic disc. Likewise, the Fovea-DPE depth was the depth between the interface of the DPE and the interface of the fovea (Fig. 2).

The location of the DPE on the coronal plane was categorized into the following 4 groups. The eyes that had the center of DPE in a 3000 μm radius of the fovea was categorized as the near fovea group. The eyes that had the center of DPE in the inferior half of the globe based on the imaginary line connecting the fovea and the center of the disc and excluded the fovea, disc area was categorized as the inferior hemisphere group. The eyes that had the center of DPE in the superior half of the globe based on the imaginary line connecting the fovea and the center of the disc and excluded the fovea, disc area was categorized as the superior hemisphere group. The eyes that had the center of DPE in a 3000 μm radius of the fovea was categorized as the near disc group (Fig. 2).

### Measurement of Optic Disc Tilt and Torsion

Digital retinal photographs centered on the optic disc were obtained using standardized settings. Optic disc torsion was measured from photographs by two observers (YCK and YJ) using the National Institutes of Health image analysis software (ImageJ 1.40; available from http://rsb.info.nih.gov/ij/index.html, National Institutes of Health, Bethesda, MD, USA). Disc torsion was identified and defined as the deviation of the long axis of the optic disc from the vertical meridian^23, 24^. The vertical meridian was considered a vertical line 90° from a horizontal line connection from the fovea, which is 2° to 6° below the optic disc, to the center of the optic disc. The angle between the vertical meridian and the longest diameter of the optic disc was considered the degree of torsion (Fig. 2). A positive torsion value indicated inferotemporal torsion (which is counterclockwise torsion in the right eye format), and a negative value indicated superonasal torsion (which is clockwise torsion in the right eye format). Details have been described previously^25^.

**Figure 3:**
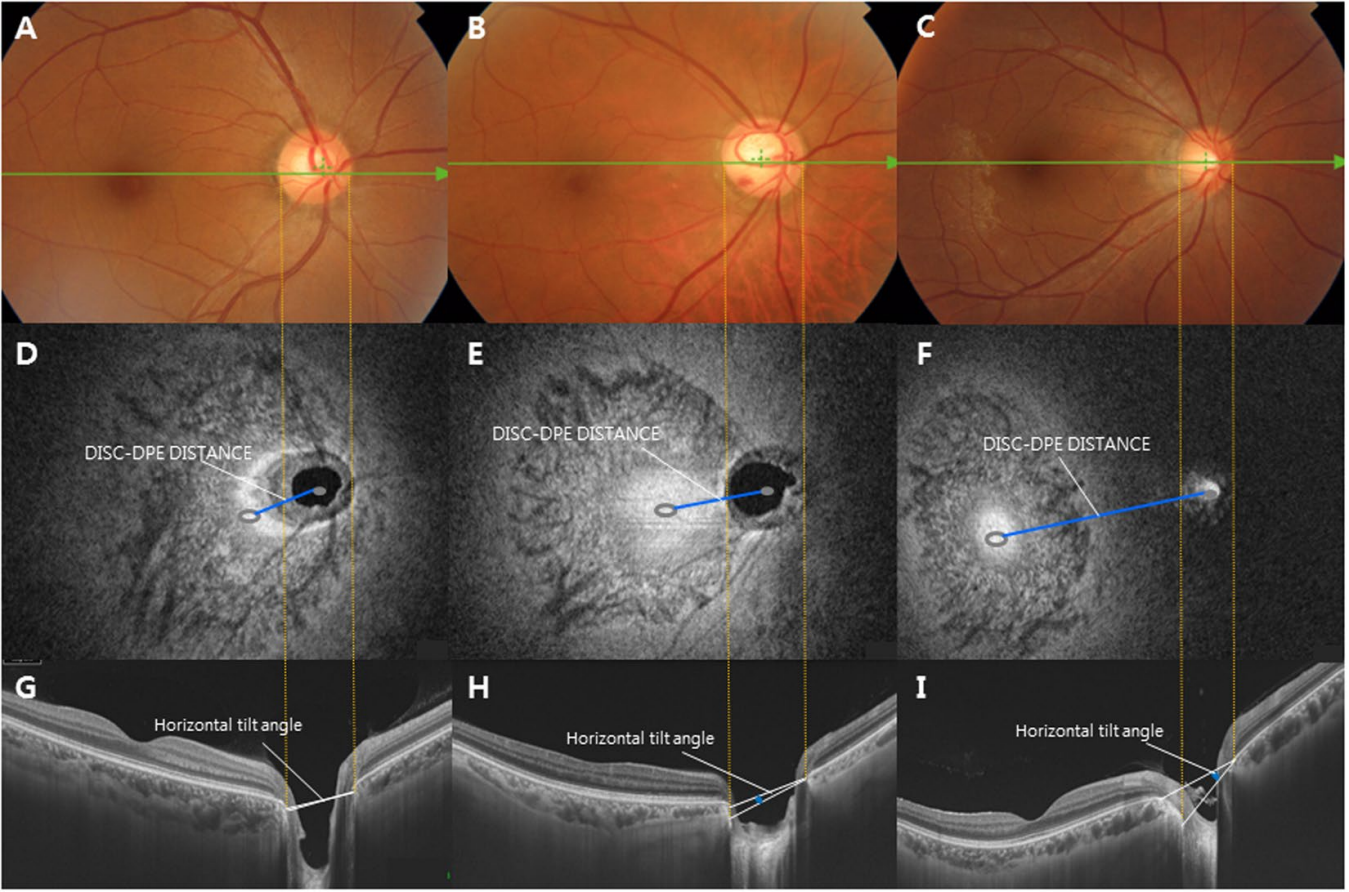
Disc photograph (**A**–**C**) and C-scan images (**D**–**F**) and B-scan images (**G**–**I**) of three different subjects with different disc to the deepest point of the eyeball (DPE) distances. As Disc-DPE distance elongated (**D**–**F**), the horizontal tilt angle significantly enlarged.

Optic disc tilt was identified by the disc ovality ratio, defined as the ratio between the longest and shortest diameters of the optic disc (tilt ratio = LD/SD)^26, 27^. Degree of tilt was also identified from SSOCT horizontal and vertical cross-sectional images. Horizontal disc tilt angle was measured according to a method proposed by Hosseini *et al*.^28^. Vertical disc tilt degree was also measured according to the method above. The horizontal scan was aligned with an imaginary line passing through the center of the disc and the fovea. A 6 mm vertical line scan 90° vertical to the horizontal scan was also obtained. Disc photographs were overlapped with 50% transparency on the En face image. The clinical disc margin points were marked using the combined disc photographs and En face images. A line connecting the two points marking the clinical disc margin on the cross-sectional images was defined as the optic nerve head (ONH) plane. A line connecting the inner tips of Bruch’s membrane on each side of the ONH plane on the cross-sectional images was drawn as the reference plane. Degree of tilt was defined as the angle between the reference plane and the ONH plane. Angle measurements were performed by two observers (YCK and HYP) with the intrinsic angle tool in the software. A positive degree of horizontal tilt indicated a temporal tilt, and a negative value indicated a nasal tilt. The degree of vertical tilt was also measured from a vertical cross-sectional image as the same way written above. A positive degree of tilt indicated an inferior tilt, and a negative value indicated a superior tilt (Fig. 3).

### Measurement Reproducibility

In the present study, the interface between the Bruch’s membrane and the choroidal tissue was determined manually by the two masked observers. Because this study is the first attempt to locate the DPE in en face images and the posterior pole contour can be altered by the direction of the globe, measurement reproducibility was required. The analysis was based on 25 independent series (10 on emmetropic eyes and 15 on myopic eyes) of intervisit reproducibility conducted twice on different days by the 2 authors (YCK and YJ). The intraclass correlation coefficients were determined by two way mixed effect model^29^. ICC scores over 0.75 are considered excellent (Table 1)^30^.

**Table 1:** Reproducibility of the measurements of the deepest point of the eyeball interface, Disc-DPE distance, Disc-DPE depth, Fovea–DPE depth, and Disc-DPE angle using swept-source optical coherence tomography in emmetropic and myopic eyes.

	Intraobserver ICC*		Interobserver ICC^†^
	ICC (Observer 1)	ICC (Observer 2)	
DPE interface	1.000 (0.999–1.000)	0.999 (0.999–1.000)	0.998 (0.994–0.999)
Disc-DPE distance, mm	0.982 (0.945–0.994)	0.974 (0.922–0.991)	0.936 (0.910–0.969)
Disc-DPE Depth, mm	0.998 (0.995–0.999)	0.998 (0.995–0.999)	0.998 (0.995–0.999)
Fovea-DPE Depth, mm	0.998 (0.995–0.999)	0.998 (0.995–0.999)	0.996 (0.992–0.999)
Disc–DPE angle (°)	0.985 (0.965–0.993))	0.973 (0.939–0.988)	0.965 (0.920–0.984)

DPE: deepest point of the eyeball; ICC: intraclass correlation coefficient.

*ICC for single measure.

^†^ICC for average measure.

### Statistical Analysis

Statistical analyses were performed using the SPSS software (version 23.0; SPSS, Chicago, IL, USA). The independent *t*-test and one way ANOVA were used to compare the data among the groups. The χ² test was used to analyze categoric variables. The Pearson’s correlation analysis was calculated to assess the relationships of the DPE quantification and the ocular parameters. Univariate and multivariate linear regression analyses were conducted to identify the association between the posterior pole profiles and disc tilt and torsion. Dependent variables were tilt and torsion, and the independent variables were Disc-DPE distance, Fovea-DPE distance, Disc-DPE depth, Fovea-DPE depth and Disc-DPE angle. The variables that retained significance at *P* < 0.2 in the univariate analysis were included in the multivariate model. The level of statistical significance was set at *P* < 0.05.

## Results

A total of 165 eyes of 165 subjects were included in the current study. Of the 165 eyes of 165 subjects, 125 eyes were classified as the myopic group and 40 eyes were classified as the emmetropic group based on the criteria of 24.0 mm axial length.

Table 2 summarizes the clinical and demographic characteristics of the enrolled subjects of the emmetropic eyes and the myopic eyes. The myopic patients were younger (59.8 ± 11.9 vs. 43.7 ± 12.0 years, respectively; *P* < 0.001), had longer axial length (23.1 ± 0.6 vs. 26.0 ± 1.3 mm, respectively; *P* < 0.001), had myopic spherical equivalent (0.2 ± 1.4 vs. −4.3 ± 3.5 D, respectively; *P* < 0.001) than the emmetropic subjects. While central corneal thickness (534.6 ± 32.1 vs. 534.6 ± 45.2 μm; respectively; *P* = 0.969) and mean deviation of perimetry (−0.95 ± 1.8 vs. −1.45 ± 1.8 dB, respectively; *P* = 0.130) was not significantly different between the emmetropic subjects and the myopic subjects, the myopic subjects had thinner RNFL than the emmetropic subjects (102.3 ± 10.7 vs. 94.4 ± 14.1 μm, respectively; *P* = 0.002).

**Table 2:** Baseline characteristics of the emmetropic eyes and the myopic eyes*.

	Emmetropic Eyes (*n* = 40)	Myopic Eyes (*n* = 125)	*P value* ^†^
Age, years	59.75 ± 11.88	43.70 ± 11.96	<**0.001** ^‡^
BCVA, logMAR	0.92 ± 0.47	1.15 ± 0.28	0.264
Laterality, right:left	26:14	55:70	**0.021** ^§^
Sex, male:female	12:28	51:74	0.221^§^
SE, diopter	0.243 ± 1.39	−4.34 ± 3.54	<**0.001** ^‡^
Axial length, mm	23.11 ± 0.62	26.02 ± 1.33	<**0.001** ^‡^
Anterior chamber length, mm	2.75 ± 0.59	3.21 ± 0.64	<**0.001** ^‡^
Central corneal thickness, μm	534.6 ± 32.08	534.6 ± 45.24	0.969
Visual field MD, dB	−0.95 ± 1.85	−1.45 ± 1.78	0.130
Average RNFL thickness, μm	102.32 ± 10.71	94.40 ± 14.09	**0.002** ^‡^

BCVA: best-corrected visual acuity; logMAR: logarithm of the minimum angle of resolution; SE: spherical equivalent; MD: mean deviation of perimetry; RNFL: retinal nerve fiber layer.

*Data are presented as mean ± standard deviation unless otherwise indicated.

^†^Independent *t*-test for continuous variables.

^‡^Statistically significant values (*P* < 0.05) are shown in bold.

^§^χ² test for categorical variables.

**Figure 4:**
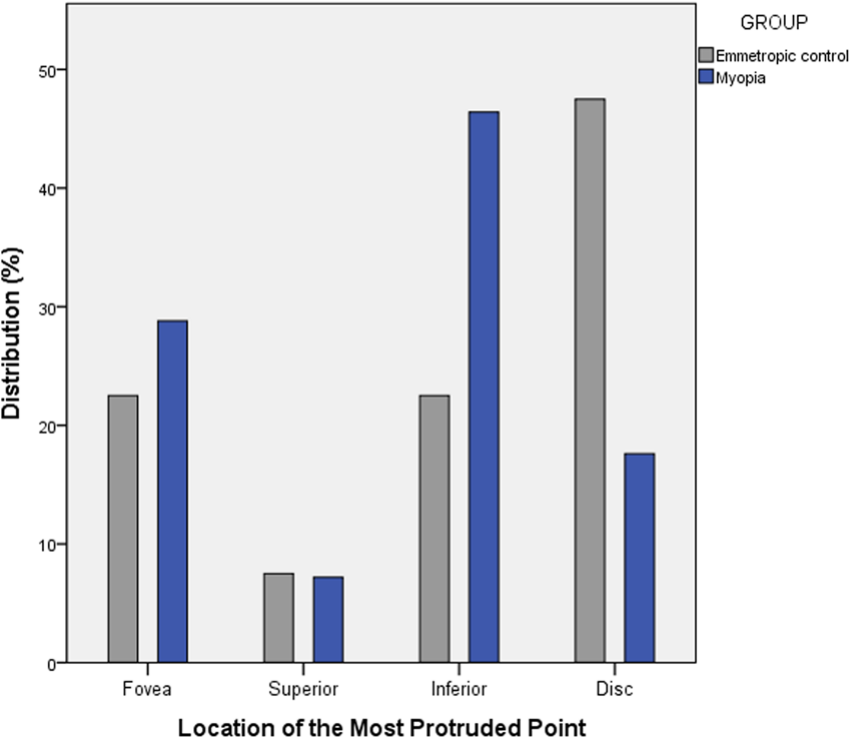
Histogram showing the distribution of the location of the most protruded point (MPP). In the emmetropic controls, the MPP was located near the optic disc (47.5%), followed by fovea, inferior, and superior (22.5%, 22.5%, and 7.5%, respectively). In the myopic group, in most eyes, the MPP was at the inferior (46.4%) followed by the fovea, disc, and superior (28.8%, 17.6%, and 7.2%, respectively). The two groups were significantly different (*P* = 0.001).

Table 3 shows the comparison of DPE and optic disc configuration between the emmetropic group and myopic group. There was significant difference in the distribution of the DPE location between the emmetropic eyes and the myopic eyes. While the location of the DPE in the emmetropic group had majority near the disc (47.5%) followed by fovea, inferior and superior (22.5%, 22.5% and 7.5%, respectively), the myopic group had majority of eyes DPE at the inferior (43.2%) followed by fovea, disc, superior (31.2%, 20.0% and 5.6%, respectively) which were significantly different between the two groups (*P* < 0.001) (Fig. 4). The Disc-DPE distance was significantly larger in the myopic eyes than the emmetropic eyes (3661.5 ± 1308.5 vs. 2667.5 ± 1103.5 μm, respectively; *P* < 0.001). The Disc-DPE depth was also significantly larger in the myopic eyes than the emmetropic eyes (128.8 ± 170.1 vs. 35.9 ± 84.4 μm, respectively; *P* = 0.001). On the other hand, the Fovea-DPE distance was significantly shorter in the myopic eyes than the emmetropic eyes (2277.8 ± 1165.4 vs. 2764.7 ± 955.4 μm, respectively; *P* = 0.019). The fovea-DPE depth was shorter in the myopic eyes than the emmetropic eyes but had no significant statistical difference (176.4 ± 172.4 vs. 355.3 ± 109.2 μm, respectively; *P* = 0.473). There was significantly larger disc torsion angle, larger disc ovality, larger horizontal tilt angle, larger vertical tilt angle in the myopic eyes than the emmetopic eyes (*P* = 0.037, *P* < 0.001, *P* < 0.001 and *P* = 0.001, respectively).

**Table 3:** Comparison of DPE properties and optic disc configuration between emmetropic and myopic eyes*.

	Emmetropic Eyes (*n* = 40)	Myopic Eyes (*n* = 125)	*P value* ^†^
Location of the DPE (eyes) Fovea: superior: inferior: disc (%)	9: 3: 9: 19 22.5: 7.5: 22.5: 47.55	39: 7: 54: 25 31.2: 5.6: 43.2: 20.0	<**0.001** ^§^
Disc-DPE distance, μm	2667.51 ± 1103.49	3661.52 ± 1308.55	<**0.001** ^‡^
Fovea-DPE distance, μm	2764.66 ± 955.36	2277.75 ± 1165.41	**0.019** ^‡^
Disc-DPE depth, μm	35.93 ± 84.49	128.81 ± 170.11	**0.001** ^‡^
Fovea-DPE depth, μm	355.26 ± 109.23	176.35 ± 172.38	0.473
Disc–fovea angle (°)	6.49 ± 3.59	7.36 ± 3.45	0.177
Disc–DPE angle (°)	20.81 ± 26.86	25.93 ± 25.81	0.286
Disc torsion angle (°)	4.41 ± 23.29	14.75 ± 27.81	**0.037** ^‡^
Disc ovality	1.11 ± 0.08	1.25 ± 0.18	<**0.001** ^‡^
Horizontal tilt angle (°)	3.23 ± 6.26	10.95 ± 10.16	<**0.001** ^‡^
Vertical tilt angle (°)	0.87 ± 2.39	5.19 ± 7.94	**0.001** ^‡^

DPE: deepest point of the eyeball.

*Data are presented as mean ± standard deviation unless otherwise indicated.

^†^Independent *t*-test for continuous variables.

^‡^Statistically significant values (*P* < 0.05) are shown in bold.

^§^χ² test for categorical variables.

Regarding the location of DPE in the myopic eyes, 39 eyes were near the fovea, 7 were in superior hemisphere, 54 were in inferior hemisphere, 25 were near the optic disc. While baseline characteristics of the 4 groups had no significant difference with each other, posterior pole profile of the Disc-DPE distance, Fovea-DPE distance, Disc-DPE depth, Fovea-DPE depth, Disc-DPE angle, disc torsion angle all had statistically significant difference (all *P* < 0.001, respectively) between the 4 groups. The group where DPE was in the inferior hemisphere had the largest Disc-DPE angle (37.3 ± 20.1°), the largest disc torsion angle (24.8 ± 26.3°), the largest vertical tilt angle (12.0 ± 8.1°) of the 4 groups (Table 4).

**Table 4:** Comparison of posterior pole characteristics according to the different locations of the DPE in myopic patients*.

	Near Fovea	Superior Hemisphere	Inferior Hemisphere	Near Disc	*P value* ^†^
Number of eyes	39	7	54	25	
Age, years	41.6 ± 12.0	49.5 ± 7.8	43.18 ± 13.5	46.4 ± 8.1	0.241
SE, diopter	−4.2 ± 3.2	−2.3 ± 1.8	−4.6 ± 4.9	−4.6 ± 4.9	0.434
Axial length, mm	26.2 ± 1.3	25.3 ± 1.2	26.1 ± 1.2	25.8 ± 1.3	0.303
Visual field MD, dB	−1.4 ± 1.6	−1.7 ± 2.2	−1.5 ± 1.9	−1.4 ± 1.9	0.980
Average RNFL thickness, μm	93.2 ± 12.9	99.1 ± 9.8	94.4 ± 13.2	94.8 ± 18.3	0.787
Disc-DPE distance, μm	4541.1 ± 584.3	3609.2 ± 953.1	3840.8 ± 1225.3	1916.8 ± 512.4	<**0.001** ^‡^
Fovea-DPE distance, μm	980.8 ± 417.7	2629.9 ± 508.4	2667.18 ± 888.0	3361.1 ± 775.7	<**0.001** ^‡^
Disc-DPE depth, μm	203.93 ± 148.9	56.4 ± 66.92	134.7 ± 161.6	19.2 ± 179.8	<**0.001** ^‡^
Fovea-DPE depth, μm	264.5 ± 48.4	316.8 ± 30.9	415.8 ± 173.2	482.2 ± 216.2	<**0.001** ^‡^
Disc–fovea angle (°)	6.4 ± 3.1	7.7 ± 3.4	7.8 ± 3.3	7.6 ± 4.2	0.265
Disc–DPE angle (°)	12.9 ± 9.4	−23.5 ± 8.1	37.3 ± 20.1	35.4 ± 31.9	<**0.001** ^‡^
Disc torsion angle (°)	7.2 ± 13.6	−21.4 ± 9.9	24.8 ± 26.3	20.2 ± 38.7	<**0.001** ^‡^
Disc ovality	1.42 ± 0.01	1.08 ± 0.02	1.22 ± 0.01	1.13 ± 0.18	<**0.001** ^‡^
Horizontal tilt angle (°)	26.8 ± 8.9	18.6 ± 9.7	17.8 ± 10.1	15.2 ± 11.3	<**0.001** ^‡^
Vertical tilt angle (°)	10.5 ± 9.7	8.6 ± 11.1	12.1 ± 10.8	9.7 ± 10.3	0.685

SE: spherical equivalent; MD: mean deviation of perimetry; RNFL: retinal nerve fiber layer; DPE: deepest point of the eyeball.

*Data are presented as mean ± standard deviation unless otherwise indicated.

^†^Kruskal–Wallis test for continuous variables between the four groups.

^‡^Statistically significant values (*P* < 0.05) are shown in bold.

**Figure 5:**
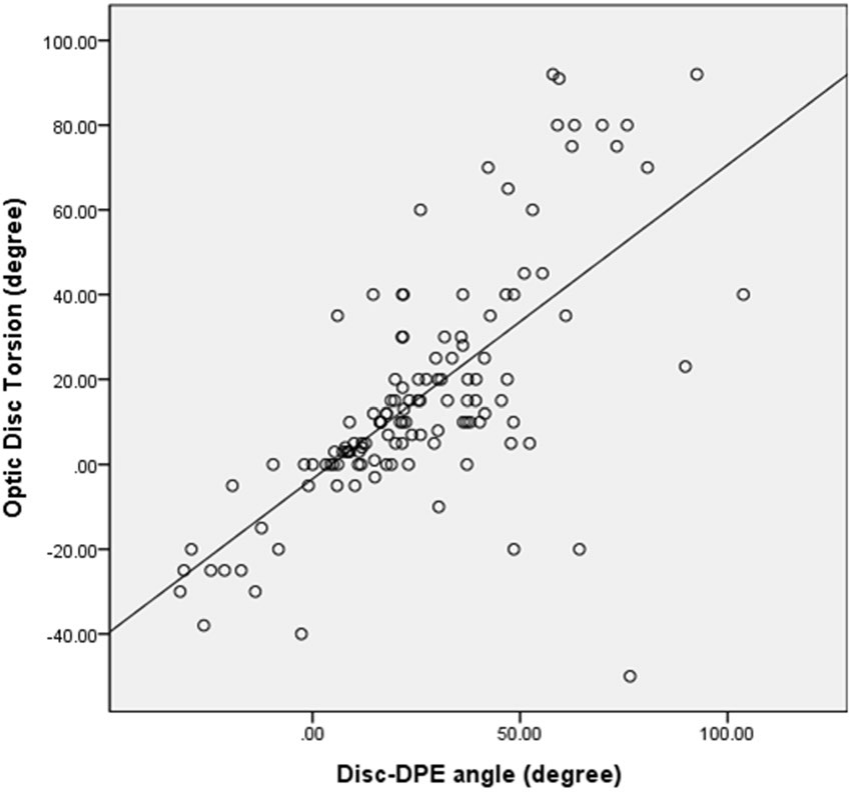
Correlation between the Disc-DPE angle and the disc torsion degree.

**Figure 6:**
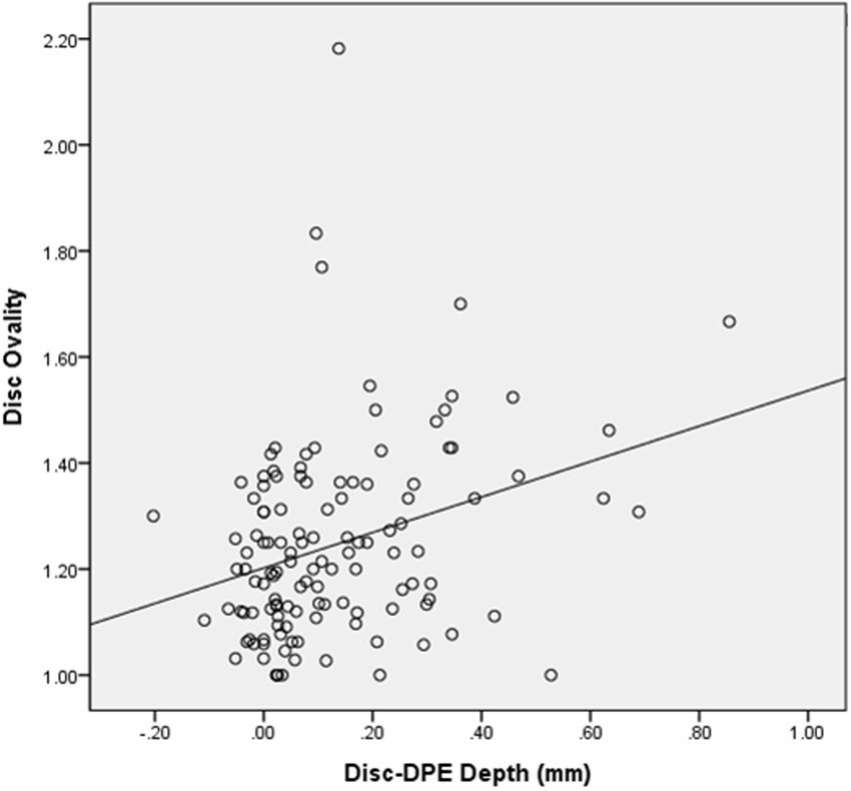
Correlation between the Disc-DPE depth and the disc ovality.

The relationship between the DPE parameters and the posterior pole characteristics was assessed in the emmetropic and myopic eyes (Table 5). The Disc-DPE distance was significantly associated with axial length (*R* = 0.314, *P* < 0.001), the Disc-DPE depth (*R* = 0.673, *P* < 0.001), disc ovality (*R* = 0.336, *P* < 0.001), degree of horizontal tilt (*R* = 0.317, *P* < 0.001) and showed a negative correlation with age, spherical equivalent, Fovea-DPE distance, Fovea-DPE depth. The Disc-DPE angle was significantly associated with disc torsion angle (*R* = 0.673, *P* < 0.001), Fovea-DPE distance (*R* = 0.517, *P* < 0.001), Fovea-DPE depth (*R* = 0.423, *P* < 0.001), and degree of vertical tilt (*R* = 0.304, *P* < 0.001) (Fig. 5). The Disc-DPE depth was significantly associated with axial length (*R* = 0.277, *P* < 0.001), the DPE to disc distance (*R* = 0.673, *P* < 0.001), disc ovality (*R* = 0.377, *P* < 0.001), degree of horizontal tilt (*R* = 0.182, *P* = 0.019) and showed a negative correlation with age, spherical equivalent, mean deviation of perimetry, average RNFL thickness and the Fovea-DPE distance as well (Fig. 6).

**Table 5:** Relationship between the posterior pole characteristics and the DPE in emmetropic and myopic eyes*.

Variables	Disc-DPE distance		Disc-DPE depth		Disc–DPE angle	
	*R*	*P* Value*	*R*	*P* Value*	*R*	*P* Value*
Age, years	−0.292	<**0.001** ^†^	−0.237	**0.002** ^†^	0.088	0.261
SE, diopter	−0.251	**0.001** ^†^	−0.260	**0.001** ^†^	−0.104	0.183
Axial length, mm	0.314	<**0.001** ^†^	0.277	<**0.001** ^†^	0.108	0.166
Visual field MD, dB	−0.110	0.161	−0.197	**0.012** ^†^	−0.152	0.052
Average RNFL thickness, μm	−0.184	0.068	−0.214	**0.006** ^†^	−0.021	0.786
Disc-DPE distance, μm			0.673	<**0.001** ^†^	−0.102	0.191
Fovea-DPE distance, μm	−0.506	<**0.001** ^†^	−0.167	0.063	0.517	<**0.001** ^†^
Disc-DPE depth, μm	0.673	<**0.001** ^†^			−0.051	0.516
Fovea-DPE depth, μm	−0.563	<**0.001** ^†^	−0.722	<**0.001** ^†^	0.423	<**0.001** ^†^
Disc–fovea angle (°)	0.015	0.846	−0.104	0.184	0.268	**0.001** ^†^
Disc–DPE angle (°)	−0.136	0.131	−0.051	0.516		
Disc torsion angle (°)	0.062	0.426	0.075	0.339	0.673	<**0.001** ^†^
Disc ovality	0.336	<**0.001** ^†^	0.377	<**0.001** ^†^	0.003	0.965
Horizontal tilt angle (°)	0.317	<**0.001** ^†^	0.182	**0.019** ^†^	0.003	0.973
Vertical tilt angle (°)	0.097	0.217	−0.051	0.512	0.304	<**0.001** ^†^

R = correlation coefficient; SE: spherical equivalent; MD: mean deviation of perimetry; RNFL: retinal nerve fiber layer; DPE: deepest point of the eyeball.

*Pearson’s correlation analysis.

^†^Statistically significant values (*P* < 0.05) are shown in bold.

Linear regression analysis was done in the emmetropic group and in the myopic group separately to compare it relationship between the optic disc configuration and the posterior pole profile. On univariate and multivariate analysis, degree of optic disc torsion increased (i.e. became more positive) with larger Disc-DPE angle (*P* < 0.001) in both emmetropic group and myopic group with the regression coefficient higher in the myopic group (β = 0.667 (0.410–0.925) vs β = 0.740 (0.597–0.882)) (Fig. 5). Moreover on univariate and multivariate analysis, degree of disc ovality increased with larger Disc-DPE depth (*P* < 0.001) in both emmetropic group and myopic group with the regression coefficient higher in the emmetropic group (β = 0.498 (0.229–0.767) vs β = 0.334 (0.150–0.519)) (Fig. 6). In the linear regression analysis on the whole subjects including both the emmetropic group and the myopic group, optic disc torsion was significantly associated with Disc-DPE angle and disc ovality was significantly associated with Disc-DPE depth as shown above (Table 6).

**Table 6:** Univariate and multivariate regression analyses of optic disc torsion and ovality index on emmetropic eyes and myopic eyes*.

	Univariate Analyses		Multivariate Analyses*		Univariate Analyses		Multivariate Analyses*	
	Beta	*P* Value	Beta (95% CI)	*P* Value	Beta	*P* Value	Beta (95% CI)	*P* Value
	**Optic Disc Torsion (Emmetropic eyes, n = 40)**				**Optic Disc Torsion (Myopic eyes, n = 125)**			
Disc-DPE distance, per 1 mm larger	−1.668	0.681			0.206	0.916		
Disc-DPE depth, per 1 mm larger	38.82	0.461			1.445	0.923		
Fovea-DPE depth, per 1 mm larger	125.34	**0.001** ^†^			31.44	**0.001** ^†^		
Disc–DPE angle, per 1° larger	0.667	<**0.001** ^†^	0.667 (0.410 to 0.925)	<**0.001** ^†^	0.740	<**0.001** ^†^	0.740 (0.597 to 0.882)	<**0.001** ^†^
	**Ovality index (Emmetropic eyes, n=40)**				**Ovality index (Myopic eyes, n=125)**			
Disc-DPE distance, per 1 mm larger	0.027	**0.021** ^†^			0.035	**0.005** ^†^		
Disc-DPE depth, per 1 mm larger	0.498	**0.001** ^†^	0.498 (0.229 to 0.767)	**0.001** ^†^	0.334	<**0.001** ^†^	0.334 (0.150 to 0.519)	<**0.001** ^†^
Fovea-DPE depth, per 1 mm larger	−0.119	0.328			−0.132	**0.042** ^†^		
Disc–DPE angle,, per 1° larger	0.000	0.936			0.000	0.737		
	**Optic Disc Torsion (Emmetropic and Myopic eyes, n = 165)**				**Ovality index (Emmetropic and Myopic eyes, n = 165)**			
Disc-DPE distance, per 1 mm larger	1.332	0.426			0.044	<**0.001** ^†^		
Disc-DPE depth, per 1 mm larger	13.39	0.339			0.417	<**0.001** ^†^	0.417 (0.258 to 0.575)	<**0.001** ^†^
Fovea-DPE depth, per 1 mm larger	30.01	**0.002** ^†^			−0.176	**0.003** ^†^		
Disc–DPE angle,, per 1° larger	0.736	<**0.001** ^†^	0.736 (0.611 to 0.861)	<**0.001** ^†^	0.000	0.965		

CI = confidence interval, DPE: deepest point of the eyeball.

*Variables with *P* < 0.05 in univariate analyses were included in multivariate analyses.

^†^Statistically significant values (*P* < 0.05) are shown in bold.

The English in this document has been checked by at least two professional editors, both native speakers of English. For a certificate, please see:

http://www.textcheck.com/certificate/GrHOaJ.

## Discussion

We describe a new method of determining the deepest point of the eyeball in human myopia. The consecutive coronal section images by ssOCT enabled us to examine the deepest point of posterior pole *in situ* in human subjects. The DPE location on the coronal section had a significant relationship with the optic disc tilt and torsion in non-glaucomatous emmetropic and myopic eyes.

**Figure 7:**
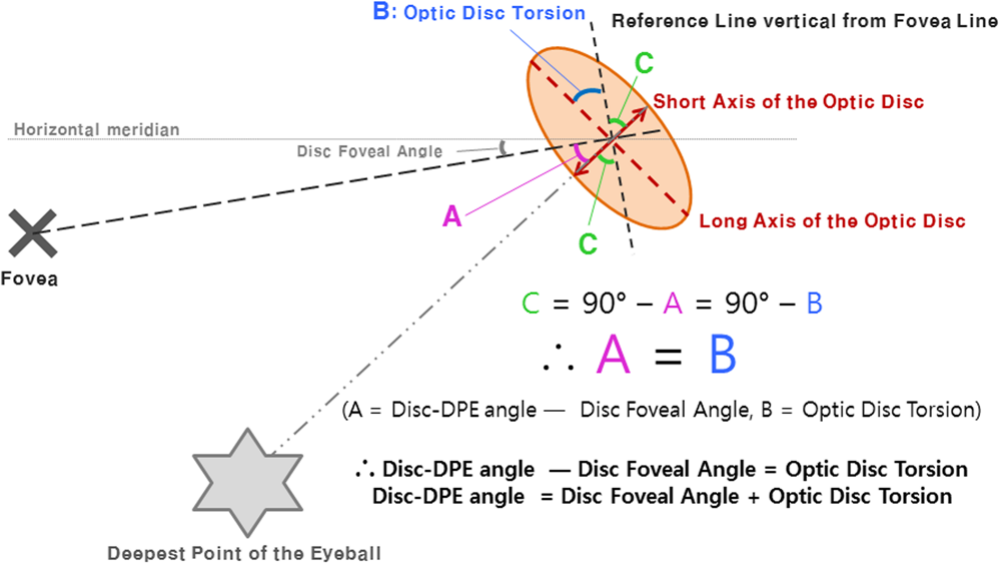
Geometric calculation on the degree of the angle of the the Disc-DPE angle as the sum of the degree of optic disc torsion and the disc foveal angle.

Our observation has implications for the pathogenesis of optic disc torsion. So far, prominent optic disc torsion has been documented in the myopic glaucomatous eyes^31^. Our data suggests that optic disc torsion degree had a strong association with the DPE location (Disc-DPE angle) on the coronal section in myopic subjects. As the axial elongation progresses, temporal border of the optic disc plane is pulled toward the DPE, resulting in an oblique plane which the temporal border locates posterior compared to the nasal border. If the structure is imaged at an oblique plane, the two-dimensional assessment of the image will lead to an underestimation of the real size^32^. Thereby, if the deepest point of the eyeball is inferior to the optic disc, the optic disc diameter will be shortest in the same angle where extension line of the DPE is located. Then, if the optic disc is close to a round circle, the long axis of the oblique optic disc would be located perpendicular to the short axis. In addition, when measuring optic disc torsion, we measure the deviation of the long axis of the optic disc from the perpendicular line from a horizontal line connection from the fovea. The four different line stated above are constituted in two right angle that shares a same center which means geometric calculation on the angle made of any of two lines out of four can be made (Fig. 7). This hypothesis is supported by our observation on the strong relationship between Disc-DPE angle and optic disc torsion angle (*R* = 0.673, *P* < 0.001). The subgroup analysis according to the different locations of the DPE revealed this geometric calculation which the Disc-DPE angle is the addition of Disc-fovea angle and the disc torsion angle. The DPE located near fovea subgroup showed a disc-foveal angle of 6.4 ± 3.1°, a disc torsion angle of 7.2 ± 13.6°, a Disc-DPE angle of 12.9 ± 9.4°, respectively. The DPE located in the superior hemisphere subgroup showed a disc-foveal angle of 7.7 ± 3.4°, a disc torsion angle of −21.4 ± 9.9°, a Disc-DPE angle of −23.5 ± 8.1°, respectively. The DPE located in the inferior hemisphere subgroup showed a disc-foveal angle of 7.8 ± 3.3°, a disc torsion angle of 24.8 ± 26.3°, a Disc-DPE angle of 37.3 ± 20.1°, respectively. A relative inaccuracy on this geometric calculation of the DPE located near disc subgroup can stand as another logic for our hypothesis. In the peripapillary sclera, the pattern of basket-woven lamellae changes into a circumferential orientation around the optic nerve head. Elastin, which is sparse in the equatorial sclera, dramatically increases in proportion near the peripapillary sclera, also running circumferentially in histologic evaluations and with imaging modalities^33, 34^. Localized elongation near the disc would make the vulnerable peripapillary tissue less predictable. Our data supports this hypothesis as this group showed larger range (−26.2° to 103.8° vs. 10° to 92.6°, −31.9° to −6.2°, −12.3° to 27.4°, respectively) and larger standard deviation (31.8° vs. 13.5°, 9.08°, 18.4°, respectively) of Disc-DPE angle opposed to other three groups.

This study has implications for the pathogenesis of optic disc tilt as well. The hypothesis on the aforementioned progressive tilting of the optic disc in children was that the optic nerve is pulled toward the temporal direction, which eventually leads the optic disc to the tilted appearance with the nasal elevation and temporal flattening^10^. Our study supports this hypothesis since the horizontal tilt angle enlarged as the distance from disc to DPE lengthened and the depth from disc to DPE deepened. Adding up to this hypothesis, optic disc tilt develops not only from the horizontal mechanical stress arising from horizontal scleral stretching at the deepest point, but also in the vertical mechanical stress arising from posterior direction scleral stretching at the DPE. The horizontal and vertical stretching stress may cause distortion in the optic nerve configuration, which might work as an additional explanation for the high risk of glaucoma in myopia^35^.

Ohno-Matsui *et al*.^36^ examined the distance and depth of most protruded point from fovea using swept source OCT. Their description of the most protruded point location is consistent with our observation that the majority of the DPE location is at the inferior part of the globe. We expanded this finding to the relationship between the deepest point of the eyeball and the optic disc configuration, as mentioned earlier. In addition, as the eyeball grew axially, the Disc-DPE distance elongated and the Disc-DPE depth deepened. Together, the axial elongation of posterior pole has a tendency to expand in the inferotemporal direction of the optic disc as the eyeball grew axially. This finding may provide a framework for understanding optic disc tilt in the temporal direction and the positive optic disc torsion in myopic subjects.

There are several limitations to the present study. First, all patients were referred to a glaucoma clinic in a tertiary hospital. We excluded myopic glaucoma patients who are more vulnerable to retinal nerve damage. Further prospective study is needed in subjects with myopic glaucoma. Second, we are unable to demonstrate conclusively that there is a relationship between axial elongation and DPE position because of the retrospective study design. The association between changes in axial length and DPE, disc change should be evaluated in future prospective studies. Third, there could be a dramatic difference in the position of the DPE once the globe rotates. Nonetheless, there was excellent reproducibility on 10 independent series of intervisit reproducibility tests conducted on different days. The reproducibility of the DPE positions can be obtained if the visual axis is fixed at the same target on each scan so none of eyeball rotation is accountable. SSOCT has real time eye tracking that has been known to eliminate the eye motion and minimizes artifacts by fixating on the fovea on each scan. Thus, like any other structures of the fundus photograph, DPE position is reproducible as long as the proper fixation is achieved. Lastly, using number of coronal sections as a depth parameter is not an accurate measurement. However, the depth calculated by number of coronal sections is an estimation to compare between different DPE of individuals. Because measuring the DPE *in vivo* in human eyes is not possible, this estimation has its significance.

The glaucoma field is at a turning point: from a two-dimensional standpoint, it is taking on three-dimensional one. A digital photograph provides two-dimensional information that records only the light intensity of the viewing object. However, the object expresses itself not only by light intensity but also by the direction of light projection. In the field of optical imaging, digital holography that expresses the direction of light projection three dimensionally will likely be commercially available in the near future. In this respect, OCT is the digital holography of the ophthalmology field. OCT not only analyzes reflected light intensity, it also analyses the direction of light projection in the target depth, using light interference^37^. The depths of the various tissues of the retina and the posterior pole can then be measured precisely. We consider that our new method of evaluating the posterior pole is one of many ways to understand the pathogenesis of myopia and glaucoma three dimensionally. We are planning the upcoming series of articles devoted to coronal section analysis of the posterior pole and its association with optic disc configuration in glaucoma patients.

In conclusion, the use of consecutive coronal section images taken by SSOCT enabled the examination of the protruded point of the posterior pole *in situ* in human subjects. The relationship between the precise location of the DPE and the optic disc tilt and torsion showed a strong correlation and may have implications in the pathogenesis of optic disc tilt and torsion.
